# Supplementary material for: Mixing indistinguishable systems leads to a quantum Gibbs paradox
Source: Nat Commun. 2021 Mar 5;12:1471. doi: 10.1038/s41467-021-21620-7 (PMC7935879; doi:10.1038/s41467-021-21620-7)
Supplement: Supplementary file 1 — Supplementary Information [file 41467_2021_21620_MOESM1_ESM.pdf]

Supplementary Information:

Mixing indistinguishable systems leads to a quantum Gibbs paradox

Yadin et al.

SUPPLEMENTARY NOTE 1 – CLASSICAL TREATMENT

Classical state space and microscopic dynamics

Here, we describe the classical setting with identical particles having an internal spin degree of freedom that is not accessed by the experimenter. The aim is to give a treatment that parallels the quantum one so that the two cases can be compared fairly. Each particle has two degree of freedom – a position $x = 1, \ldots, d$ and a spin $s = 1, \ldots, S$ – which are the accessible and hidden degrees of freedom, respectively. (Note that we only require $S = 2$ in the main text.)

We start from the point of view of a hypothetical observer for whom *all* the particles are fully distinguishable. The effective indistinguishability of the particles will be imposed later by a suitable restriction on the allowed operations. This is rather like the first-quantised description of quantum identical particles. The underlying state space of N distinguishable particles is

$$\Sigma_N = \{(\boldsymbol{x}, \boldsymbol{s}) \mid \boldsymbol{x} \in [d]^N, \boldsymbol{s} \in [S]^N\}, \tag{1}$$

where $[k] = \{1, 2, \ldots, k\}$. This can be expressed as a Cartesian product $\Sigma_N = \Sigma_N^x \times \Sigma_N^s$ of the individual spaces for each degree of freedom.

A thermodynamical operation involves coupling the particles to a heat bath and work reservoir, the latter two of which we group into a joint system called the "apparatus" A. This has its own state space Σ_A whose states we designate by a label a. A state of the whole system can therefore be specified by a tuple $(\boldsymbol{x}, \boldsymbol{s}, a)$. We assume the underlying microscopic dynamics to be deterministic and reversible; thus, an evolution of whole system consists of an invertible mapping

$$(\boldsymbol{x}, \boldsymbol{s}, a) \to (\boldsymbol{x}', \boldsymbol{s}', a'). \tag{2}$$

Dynamics independent of spin and particle label

Now we impose the condition that the operation be spin independent. This translates into two features: i) the spins are all unchanged, so $\boldsymbol{s}' = \boldsymbol{s}$, and ii) $\boldsymbol{x}'$ and a' are functions of $\boldsymbol{x}$ and a only, not $\boldsymbol{s}$. It is clear that $\boldsymbol{s}$ is completely decoupled from the other variables, so that the dynamics of the apparatus are the same for any value of $\boldsymbol{s}$. Thus we can drop the redundant information and designate states of the whole system by $(\boldsymbol{x}, a)$.

Next, we impose operational indistinguishability of the particles, again by restricting the allowed operations. An allowed operation must be invariant under a rearrangement of particle labels. For a permutation $\pi \in S_N$, let $\pi[\boldsymbol{x}] = (x_{\pi(1)}, \ldots, x_{\pi(N)})$. Then we require that

$$(\boldsymbol{x}, a) \to (\boldsymbol{x}', a') \Rightarrow (\pi[\boldsymbol{x}], a) \to (\pi[\boldsymbol{x}'], a') \quad \forall \pi \in S_N, \tag{3}$$

i.e., the transformation commutes with all permutations. This condition implies that a' is a function only of a and the *type* $\boldsymbol{t}$ of $\boldsymbol{x}$. By this, we mean $\boldsymbol{t} = (t_1, \ldots, t_d)$ specifies the number t_i of particles in each cell i. It is then clear that, as far as the dynamics of A are concerned, it is sufficient to keep track of just $(\boldsymbol{t}, a)$. The total number of effective microstates of the particles, as seen by the ignorant observer, is then the number of possible types, equal to $\binom{N+d-1}{N}$.

Subtlety with overly constrained dynamics

However, there is a subtlety: one can ask whether all (deterministic and reversible) dynamics in the space of $(\boldsymbol{t}, a)$ are possible under the constraint Supplementary Eq. (3). If $(\boldsymbol{t}, a) \to (\boldsymbol{t}', a')$ is possible, then there exist some $\boldsymbol{x}, \boldsymbol{x}'$ of types $\boldsymbol{t}, \boldsymbol{t}'$ respectively such that $(\boldsymbol{x}, a) \to (\boldsymbol{x}', a')$. The condition Supplementary Eq. (3) then determines how all the remaining vectors $\pi[\boldsymbol{x}]$ of type $\boldsymbol{t}$ evolve. There may be a contradiction here – there are two ways in which a transformation might not be possible:

- If there exists π such that $\pi[\boldsymbol{x}] = \boldsymbol{x}$ but $\pi[\boldsymbol{x}'] \neq \boldsymbol{x}'$, then the transformation cannot be deterministic.
- If there exists π such that $\pi[\boldsymbol{x}] \neq \boldsymbol{x}$ but $\pi[\boldsymbol{x}'] = \boldsymbol{x}'$, then the transformation cannot be reversible.

We give the following example, consider $\boldsymbol{x} = (1, 1)$, $\boldsymbol{x}' = (1, 2)$, which have types $\boldsymbol{t} = (2, 0)$, $\boldsymbol{t}' = (1, 1)$. A swap of the two particles preserves $\boldsymbol{x}$ but not $\boldsymbol{x}'$ – it is clear that a transition $\boldsymbol{t} \to \boldsymbol{t}'$ cannot be possible. In other words,

this is because there is no way of "picking out" a particle from cell 1 and moving it to cell 2 in a way that acts non-preferentially on the particles. In quantum mechanics, this obstacle is avoided because it is possible to act symmetrically on the particles such that the final state is an equal superposition of the two $\boldsymbol{x'} = (1, 2)$ and $(2, 1)$.

This hints at a way to avoid the problem in the classical case: widening the scope to include stochastic operations. Since it is crucial to require that all dynamics are microscopically deterministic, we introduce stochasticity using additional degrees of freedom containing initial randomness. These couple to the different ways the particles can be permuted, and must necessarily be *hidden*, i.e., not accessible to the observer, in order to maintain ignorance about the particle labels. The idea is to construct globally deterministic, reversible dynamics such that tracing out the hidden degrees of freedom gives stochastic dynamics on $(\boldsymbol{x}, a)$ via the probabilities $p(\boldsymbol{x}', a'|\boldsymbol{x}, a)$. Analogously to Supplementary Eq. (3), we impose the condition

$$p(\boldsymbol{x'}, a'|\boldsymbol{x}, a) = p(\pi[\boldsymbol{x'}], a'|\pi[x], a) \quad \forall \pi \in S_N. \tag{4}$$

The claim is that such dynamics exist that enable all possible (deterministic, reversible) transformations of $(\boldsymbol{t}, a)$. To see this, consider just one desired transformation $(\boldsymbol{t}, a) \to (\boldsymbol{t'}, a')$. We introduce two sets of additional variables $\boldsymbol{h_1}, \boldsymbol{h_2}$ which respectively contain information about $\boldsymbol{x}$ and $\boldsymbol{x'}$. $\boldsymbol{h_1}$ starts in a "ready" state $\boldsymbol{0}$, while $\boldsymbol{h_2}$ is uniformly distributed over all $\boldsymbol{x}'$ of type $\boldsymbol{t'}$. Writing a joint state of all subsystems as $(\boldsymbol{x}, a, \boldsymbol{h_1}, \boldsymbol{h_2})$, it is easily verified that the following dynamics are deterministic and reversible:

$$(\boldsymbol{x}, a, \boldsymbol{0}, \boldsymbol{x'}) \to (\boldsymbol{x'}, a', \boldsymbol{x}, \boldsymbol{x'}) \quad \forall \boldsymbol{x}, \boldsymbol{x'} \text{ of types } \boldsymbol{t}, \boldsymbol{t}', \tag{5}$$

where a' is of course a function of $\boldsymbol{t}$ only. Here, $\boldsymbol{h_1}$ keeps a record of the initial configuration (to ensure reversibility) and $\boldsymbol{h_2}$ randomises the final configuration to range uniformly over all $\boldsymbol{x'}$ of type $\boldsymbol{t'}$. Hence we see that $p(\boldsymbol{x}', a'|\boldsymbol{x}, a)$ is constant over all $\boldsymbol{x}, \boldsymbol{x'}$ of interest and thus satisfies condition Supplementary Eq. (4).

Note that $\boldsymbol{h_1}$ has to be initialised in a "pure" state of zero entropy such that it can record information. Such a state, being non-thermal, should be regarded as an additional resource which costs work to prepare. (By contrast, the uniformly random variable $\boldsymbol{h_2}$ is thermal and thus free.) The necessary leakage of information into $\boldsymbol{h_1}$ therefore entails dissipation of work into heat. Hence the work extraction formula (2,3)[main text] is technically an upper bound to what can be achieved classically.

This record of information about the initial configuration is seen to be necessary only for those transitions where the set of $\boldsymbol{x}$ of type $\boldsymbol{t}$ is smaller than the set of $\boldsymbol{x'}$ of type $\boldsymbol{t'}$, in order to prevent irreversible merging of states. This situation can be avoided, for instance, in the case of the classical analogue of fermions wherein no more than one particle can occupy a cell. Similarly, in the low density limit (discussion of which appears in the main text), almost all configurations are of this type with very high probability. (One could also argue that this problem is never encountered in reality – as soon as two particles overlap sufficiently, we are already in the quantum parameter regime.)

To summarise what we have shown in this section:

- Classical identical particles can be treated, analogously to the quantum case, as (in principle) distinguishable particles whose dynamics are restricted to be independent of particle label.
- An observer with access only to spin-independent operations can treat the system as if the particles were spin-less.
- There is a subtlety with the particle-label-independent operations that blocks certain transitions. This restriction can be lifted with additional degrees of freedom but may require dissipation of work into heat. This extra cost is zero when particles always occupy distinct cells.

SUPPLEMENTARY NOTE 2 – DETAILS FOR QUANTUM IGNORANT OBSERVER

In this section, we provide additional details for the entropy change as seen by the ignorant observer.

Recall that Schur-Weyl duality [1, Chapter 5] provides the decomposition

$$\mathcal{H}_x^{\otimes N} = \bigoplus_\lambda \mathcal{H}_x^\lambda \otimes \mathcal{K}_x^\lambda, \tag{6}$$

where λ runs over all Young diagrams containing N boxes and no more than d rows. A Young diagram λ is a set of unlabelled boxes arranged in rows, with non-increasing row length from top to bottom. We can equivalently describe

$\lambda = (\lambda_1, \lambda_2, \ldots, \lambda_d)$, where λ_i is the number of boxes in row i. For example, 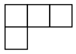 would be denoted $(3, 1)$ (where $N = 4, d = 2$).

$\mathcal{H}_x^\lambda$ and $\mathcal{K}_x^\lambda$ carry irreps of $\mathrm{U}(d)$ and S_N respectively, corresponding to irreducible subspaces under the actions of single-particle unitary rotations $u^{\otimes N} \otimes I^{\otimes N}$ and particle label permutations $\Pi \otimes I^{\otimes N}$, each of which act only on the spatial part. The same decomposition works for the spin part $\mathcal{H}_s^{\otimes N}$, although now the Young diagrams λ have maximally two rows. In fact, they correspond to the familiar SU(2) irreps with total angular momentum J, via $\lambda = (N/2 + J,\, N/2 - J)$.

After putting the spatial and spin decompositions together, projecting onto the overall (anti-)symmetric subspace causes the symmetries of the two components to be linked. For bosons, the overall symmetric subspace (itself a trivial irrep of S_N) occurs exactly once in $\mathcal{K}_x^\lambda \otimes \mathcal{K}_s^{\lambda'}$ if and only if $\lambda = \lambda'$, and otherwise does not [2, Section 7-13]. Thus we have

$$\mathcal{H}_N = \bigoplus_{\lambda,\lambda'} \mathcal{H}_x^\lambda \otimes \mathcal{H}_s^{\lambda'} \otimes P_+ \left[\mathcal{K}_x^\lambda \otimes \mathcal{K}_s^{\lambda'}\right] \tag{7}$$

$$= \bigoplus_{\lambda} \mathcal{H}_x^\lambda \otimes \mathcal{H}_s^{\lambda} \quad \text{(bosons)}. \tag{8}$$

For fermions, the only difference is that the projector P_- onto the antisymmetric subspace enforces $\lambda' = \lambda^T$, denoting the transpose of the Young diagram in which rows and columns are interchanged; thus,

$$\mathcal{H}_N = \bigoplus_{\lambda} \mathcal{H}_x^{\lambda^T} \otimes \mathcal{H}_s^{\lambda} \quad \text{(fermions)}. \tag{9}$$

Due to the use of a two-dimensional spin, we employ the correspondence $J \leftrightarrow \lambda = (N/2 + J,\, N/2 - J, 0, 0, \ldots)$ (with a total of d rows) to replace the label λ by J.

Let us first consider the bosonic case. Thanks to the decomposition in Supplementary Eq. (8), a state ρ (as seen by the informed observer) can be written in terms of the basis $|J,q\rangle_x |J,M\rangle_s |\phi_J\rangle_{xs}$, where $|J,q\rangle \in \mathcal{H}_x^J$, $|J,M\rangle \in \mathcal{H}_s^J$, $|\phi_J\rangle \in \mathcal{K}_x^J \otimes \mathcal{K}_s^J$, as described in the main text. The ignorant observer sees the reduced state after tracing out the spin part, of the form

$$\rho_x = \mathrm{tr}_s\, \rho = \bigoplus_J p_J \rho_x^J \otimes \mathrm{tr}_s\, |\phi_J\rangle\langle\phi_J|_{xs}. \tag{10}$$

The entropy of this state is

$$S(\rho_x) = H(\boldsymbol{p}) + \sum_J p_J \left[S\left(\rho_x^J\right) + S\left(\mathrm{tr}_s\, |\phi_J\rangle\langle\phi_J|_{xs}\right)\right], \tag{11}$$

where $H(\boldsymbol{p}) := -\sum_J p_J \ln p_J$ is the Shannon entropy of the probability distribution p_J.

As argued in the main text, the fully thermalised final state is of the form

$$\rho_x' = \bigoplus_J p_J \frac{I_x^J}{d_J} \otimes \mathrm{tr}_s\, |\phi_J\rangle\langle\phi_J|_{xs}, \tag{12}$$

with entropy

$$S(\rho_x') = H(\boldsymbol{p}) + \sum_J p_J \left[\ln d_J + S\left(\mathrm{tr}_s\, |\phi_J\rangle\langle\phi_J|_{xs}\right)\right]. \tag{13}$$

An example of a channel that achieves the mapping from ρ_x to ρ_x' – albeit without a coupling to a heat bath or work reservoir – is the so-called "twirling" operation. This is a probabilistic average over all single-particle unitary rotations $u_x^{\otimes N}$:

$$\mathcal{T}_x(\rho) = \int \mathrm{d}\mu(u_x)\, u_x^{\otimes N} \rho u_x^{\otimes N\dagger}, \tag{14}$$

where μ is the Haar measure over the group U(d).

The entropy change for the ignorant observer is therefore

$$\Delta S_{\text{igno}} = S(\rho_x') - S(\rho_x) = \sum_J p_J \left[\ln d_J - S\left(\rho_x^J\right)\right]. \tag{15}$$

(Note that the states ϕ_J do not enter into the entropy change.) Our goal is therefore to determine the probabilities p_J, dimensions d_J, and the entropy of the component states ρ_x^J.

The case of indistinguishable gases is dealt with in the main text: the state is fully in the subspace $J = N/2$, corresponding to the spatially symmetric subspace for bosons and spatially antisymmetric for fermions.

For gases of different spins, the initial state is such that all particles on the left are in $|\uparrow\rangle$ and all on the right are in $|\downarrow\rangle$. Before getting to the thermal state, first consider a pure state in which n_i particles are in each cell i on the left, and m_i in each cell i on the right (such that $\sum_i n_i = n$, $\sum_i m_i = m$). This spatial configuration is denoted by the pair of vectors $(\boldsymbol{n}, \boldsymbol{m})$. The properly symmetrised wavefunction is

$$|\psi(\boldsymbol{n},\boldsymbol{m})\rangle = \mathcal{N}(\boldsymbol{n},\boldsymbol{m}) \sum_{\text{distinct } \pi\in S_N} \pi|\boldsymbol{n},\boldsymbol{m}\rangle_x \otimes \pi|\uparrow^n\downarrow^m\rangle_s, \tag{16}$$

$$|\boldsymbol{n},\boldsymbol{m}\rangle := |1_L^{n_1} 2_L^{n_2} \dots 1_R^{m_1} 2_R^{m_2} \dots\rangle\,, \tag{17}$$

where π runs over permutations of the N particles that lead to *distinct* terms $\pi|\boldsymbol{n},\boldsymbol{m}\rangle_x$. (This is well-defined, since whenever π and π' have the same effect on $|\boldsymbol{n},\boldsymbol{m}\rangle$, they must also have the same effect on $|\uparrow^n\downarrow^m\rangle$.) $\mathcal{N}(\boldsymbol{n},\boldsymbol{m})$ is a normalisation factor (such that $\mathcal{N}(\boldsymbol{n},\boldsymbol{m})^{-2}$ is the number of distinct terms in the sum). We determine the p_J via the expectation value of the projector P_s^J onto the subspace $\mathcal{H}_s^J$:

$$\langle\psi(\boldsymbol{n},\boldsymbol{m})|P_s^J|\psi(\boldsymbol{n},\boldsymbol{m})\rangle \tag{18}$$

$$= \mathcal{N}(\boldsymbol{n},\boldsymbol{m})^2 \sum_{\text{distinct } \pi,\pi'} \langle\boldsymbol{n},\boldsymbol{m}|\pi'\pi|\boldsymbol{n},\boldsymbol{m}\rangle \, \langle\uparrow^n\downarrow^m|\pi' P_s^J \pi|\uparrow^n\downarrow^m\rangle \tag{19}$$

$$= \mathcal{N}(\boldsymbol{n},\boldsymbol{m})^2 \sum_{\text{distinct } \pi} \langle\uparrow^n\downarrow^m|\pi P_s^J \pi|\uparrow^n\downarrow^m\rangle\,, \tag{20}$$

where the second line holds because any pair of π, π' giving rise to distinct terms in Supplementary Eq. (17) also have different actions on $|\boldsymbol{n},\boldsymbol{m}\rangle$. Now we use Clebsch-Gordan coefficients to evaluate each term in this last sum. First note that we can express $|\uparrow^n\rangle$ as a combined spin with $J_1 = M_1 = n/2$, and similarly $|\downarrow^m\rangle$ as a spin with $J_2 = -M_2 = m/2$. The Clebsch-Gordan coefficient $C(\frac{n}{2}, \frac{n}{2}; \frac{m}{2}, \frac{-m}{2}; J, \frac{n-m}{2})$ is precisely the amplitude for this state in the J subspace. This is unchanged by the inclusion of a permutation π, so Supplementary Eq. (20) simplifies to

$$\langle\psi(\boldsymbol{n},\boldsymbol{m})|P_s^J|\psi(\boldsymbol{n},\boldsymbol{m})\rangle = \left| C\left(\frac{n}{2}, \frac{n}{2}; \frac{m}{2}, \frac{-m}{2}; J, \frac{n-m}{2}\right)\right|^2. \tag{21}$$

Now it remains to consider the correct initial state, which is a uniform probabilistic mixture of all $|\psi(\boldsymbol{n},\boldsymbol{m})\rangle$ with a fixed number of particles n, m on the left and right, respectively. Since the Clebsch-Gordan coefficient is the same for all such configurations, we have [3]

$$p_J = \left| C\left(\frac{n}{2}, \frac{n}{2}; \frac{m}{2}, \frac{-m}{2}; J, \frac{n-m}{2}\right)\right|^2 \tag{22}$$

$$= \frac{(2J+1)n!m!}{\left(\frac{N}{2}+J+1\right)!\left(\frac{N}{2}-J\right)!}. \tag{23}$$

Finally, we determine the entropy of each ρ_x^J component. Using the basis $|J,q\rangle_x |J,M\rangle_s |\phi_J\rangle_{xs}$ provided by the Schur-Weyl decomposition, we have

$$|\psi(\boldsymbol{n},\boldsymbol{m})\rangle = \sum_J \sqrt{p_J}|\psi(\boldsymbol{n},\boldsymbol{m},J)\rangle_x \left|J, \frac{n-m}{2}\right\rangle_s |\phi_J\rangle_{xs}. \tag{24}$$

Here, $|\psi(\boldsymbol{n},\boldsymbol{m},J)\rangle_x \in \mathcal{H}_x^J$ is some linear combination of the $|J,q\rangle_x$ – without needing to determine these states entirely, it will be sufficient to note that they are orthogonal for different configurations $(\boldsymbol{n},\boldsymbol{m})$. This follows from the fact that different $|\psi(\boldsymbol{n},\boldsymbol{m})\rangle$ are fully distinguishable just by measuring the occupation numbers of different cells. Tracing out s, we find

$$\mathrm{tr}_s\, \psi(\boldsymbol{n},\boldsymbol{m}) = \bigoplus_J p_J \psi(\boldsymbol{n},\boldsymbol{m},J) \otimes \mathrm{tr}_s\, |\phi_J\rangle\langle\phi_J|_{xs}, \tag{25}$$

$$\rho_x^J \propto \sum_{\boldsymbol{n},\boldsymbol{m}} \psi(\boldsymbol{n},\boldsymbol{m},J). \tag{26}$$

From orthogonality of the $\psi(\boldsymbol{n},\boldsymbol{m},J)$, it follows that

$$S(\rho_x^J) = \ln \binom{n+d/2-1}{n} + \ln \binom{m+d/2-1}{m}. \tag{27}$$

Inserted into Supplementary Eq. (15), this results in the claimed entropy changes (11,12)[main text].

SUPPLEMENTARY NOTE 3 – PARTIAL DISTINGUISHABILITY

Here, we extend the analysis to include non-orthogonal spins states. As before, we keep the initial spins on the left side of the box as $|{\uparrow}\rangle^{\otimes n}$, but now on the right we have $|{\nearrow}\rangle^{\otimes m}$, where $|{\nearrow}\rangle = \cos(\theta/2)\,|{\uparrow}\rangle + \sin(\theta/2)\,|{\downarrow}\rangle$.

Informed observer

Let us first discuss the operations allowed to be performed by the informed observer. They are permitted to know about the value of the spins in the $|{\uparrow}\rangle, |{\downarrow}\rangle$ basis; they may engineer dynamics diagonal in this basis. Of course, this choice entails a preferred spin basis – this is necessary in order to have a well-defined notion of conditioning dynamics on the value of a spin. We thus require a global unitary of the form $U = \bigoplus_M U^{(M)}_{xsBW}$, where the block structure refers to subspaces with fixed M as defined by the Schur basis. Under a block-diagonal operation, one cannot extract work from coherences between the blocks [4, 5]; that is, the initial state of the spins can be effectively replaced by the dephased state

$$\Phi(\rho_{xs}) := \sum_M Q_s^M \rho_{xs} Q_s^M = \sum_M q_M \rho_{xs}^{(M)}, \tag{28}$$

where Q_s^M is the projector onto the M block. In other words, the state behaves thermodynamically as a statistical mixture of the different z-spin numbers M. It follows that the overall entropy change is the average

$$\Delta S_{\text{info}}(\rho_{xs}) = \sum_M q_M \Delta S_{\text{info}}(\rho_{xs}^{(M)}). \tag{29}$$

As for the case of orthogonal spins, the initial state is a uniform mixture of states generalising equation Supplementary Eq. (17),

$$|\psi(\boldsymbol{n},\boldsymbol{m})\rangle = \mathcal{N}(\boldsymbol{n},\boldsymbol{m}) \sum_{\text{distinct } \pi\in S_N} \pi|\boldsymbol{n},\boldsymbol{m}\rangle_x \otimes \pi|{\uparrow}^n{\nearrow}^m\rangle_s, \tag{30}$$

where again it is sufficient (and well-defined) for π to run only over permutations that lead to distinct $\pi|\boldsymbol{n},\boldsymbol{m}\rangle_x$. As before, $\mathcal{N}^{-2}$ is simply the number of such distinct terms (independent of θ).

Expanding $|{\nearrow}^m\rangle$ in the preferred basis, it is easily seen that

$$|{\nearrow}^m\rangle = \sum_{k=0}^m \cos(\theta/2)^{m-k} \sin(\theta/2)^k \sum_{\text{distinct } \pi\in S_m} \pi\,|{\uparrow}^{m-k}{\downarrow}^k\rangle, \tag{31}$$

and so

$$q_M = \langle {\uparrow}^n{\nearrow}^m|Q_s^M|{\uparrow}^n{\nearrow}^m\rangle = \binom{m}{(n+m)/2-M} \cos(\theta/2)^{m-n+2M} \sin(\theta/2)^{n+m-2M}, \tag{32}$$

having used $M = (n+m)/2 - k$. Without needing to know the form of $Q_s^M\,|\psi(\boldsymbol{n},\boldsymbol{m})\rangle$, it is sufficient to note that all such states are pure and must be orthogonal, since they can be mutually perfectly distinguished by measuring the occupation number in each cell. The entropy $S(\rho_{xs}^{(M)})$ is therefore just as in Supplementary Eq. (27) for each M.

Due to the block-diagonal structure of the global unitary U, the maximum entropy final state is given by a maximally mixed state for each M block. Considering the number of possible spatial configurations for a fixed number of up

and down spins, the dimension of the M block is found to be $\binom{(n+m)/2-M+d-1}{(n+m)/2-M}\binom{(n+m)/2+m+d-1}{(n+m)/2+M}$ in the bosonic case. Hence the overall entropy change is

$$\Delta S_{\text{info}} = \sum_{M=-N/2}^{N/2} q_M \left[\ln\binom{N/2-M+d-1}{N/2-M} + \ln\binom{N/2+M+d-1}{N/2+M}\right] - \left[\ln\binom{n+d/2-1}{n} + \ln\binom{m+d/2-1}{m}\right]. \tag{33}$$

In the fermionic case, analogous counting gives

$$\Delta S_{\text{info}} = \sum_{M=-N/2}^{N/2} q_M \left[\ln\binom{d}{N/2-M} + \ln\binom{d}{N/2+M}\right] - \left[\ln\binom{d/2}{n} + \ln\binom{d/2}{m}\right]. \tag{34}$$

Ignorant observer

For the ignorant observer, we now have to analyse ρ_x^J. From Supplementary Eq. (30) (recalling that the permutations to be summed over are those that lead to distinct $\pi|\boldsymbol{n},\boldsymbol{m}\rangle_x$),

$$\mathrm{tr}_s\left[P_s^J\,|\psi(\boldsymbol{n},\boldsymbol{m})\rangle\langle\psi(\boldsymbol{n},\boldsymbol{m})|\right] = \mathcal{N}^2\sum_{\pi,\pi'}\langle\uparrow^n\nearrow^m|\pi^\dagger P_s^J\pi'|\uparrow^n\nearrow^m\rangle\,\pi'\,|\boldsymbol{n},\boldsymbol{m}\rangle\langle\boldsymbol{n},\boldsymbol{m}|\,\pi^\dagger \tag{35}$$

$$= \mathcal{N}^2\sum_{\pi,\pi'}\langle\uparrow^n\nearrow^m|P_s^J\pi^\dagger\pi'|\uparrow^n\nearrow^m\rangle\,\pi'\,|\boldsymbol{n},\boldsymbol{m}\rangle\langle\boldsymbol{n},\boldsymbol{m}|\,\pi^\dagger, \tag{36}$$

using the fact that the projector P_s^J commutes with permutations. In order to simplify this, we examine coefficients of the form $\langle\uparrow^n\nearrow^m|P_s^J\pi|\uparrow^n\nearrow^m\rangle$. Using the Schur basis just for the spin part, in general one can expand $|\uparrow^n\nearrow^m\rangle = \sum_{J,M,r}\omega_{J,M,r}\,|J,M,r\rangle$. The r label, representing the part of the basis acted upon by the permutation group, consists of any quantum numbers needed to complete the set along with J and M. We describe a convenient choice of such numbers, denoted $j_1, j_{1,2}, \ldots, j_{1,\ldots,n}$ and $k_1, k_{1,2}, \ldots, k_{1,\ldots,m}$. j_1 is the total spin eigenvalue of spin 1, $j_{1,2}$ of spins 1 and 2 together, and so on. $k_1, \ldots$ have the same meaning, but for the remaining spins label $n+1,\ldots,n+m$. That these complete the set of quantum numbers is evident from imagining performing an iterated Clebsch-Gordan procedure. This would involve coupling spins 1 and 2, then adding in spin 3, and so on up to spins n. Spins $n+1$ up to $n+m$ would be coupled recursively in the same manner, and then finally the two blocks of spins coupled to give the overall J.

For the state $|\uparrow^n\nearrow^m\rangle$ each of the two blocks of spins is fully symmetric, meaning that each of these spin eigenvalues is maximal: $j_1 = k_1 = \frac{1}{2}$, $j_2 = k_2 = 1$, …, $j_{1,\ldots,n} = \frac{n}{2}$, $k_{1,\ldots,m} = \frac{m}{2}$. Given this choice of basis, there is only a single value of $r = r_0$ in the expansion of $|\uparrow^n\nearrow^m\rangle$, referring to this collection of spin eigenvalues. Therefore we can write $|\uparrow^n\nearrow^m\rangle = \sum_{J,M}\omega_{J,M}\,|J,M,r_0\rangle$, and

$$\langle\uparrow^n\nearrow^m|P_s^J\pi|\uparrow^n\nearrow^m\rangle = \sum_{M,M'}\omega_{J,M'}^*\omega_{J,M}\,\langle J,M',r_0|\pi|J,M,r_0\rangle \tag{37}$$

$$= \sum_M |\omega_{J,M}|^2\,\langle J,M,r_0|\pi|J,M,r_0\rangle \tag{38}$$

$$=: \sum_M |\omega_{J,M}|^2\eta_J(\pi) \tag{39}$$

since $\langle J,M,r_0|\pi|J,M,r_0\rangle$ is independent of M (and r_0 is fixed anyhow). Expanding $|\uparrow^n\nearrow^m\rangle$ in the preferred basis and using the Clebsch-Gordan coefficients for coupling the two blocks of spins gives

$$|\omega_{J,M}|^2 = q_M\left|C\left(\frac{n}{2},\frac{n}{2};\frac{m}{2},M-\frac{n}{2};J,M\right)\right|^2, \tag{40}$$

where q_M is defined in Supplementary Eq. (32). Putting this into Supplementary Eq. (36), we have

$$\mathrm{tr}_s\left[P_s^J\,|\psi(\boldsymbol{n},\boldsymbol{m})\rangle\langle\psi(\boldsymbol{n},\boldsymbol{m})|\right] = \mathcal{N}^2\sum_{\pi,\pi'}\sum_M |\omega_{J,M}|^2\eta_J(\pi^\dagger\pi')\pi'\,|\boldsymbol{n},\boldsymbol{m}\rangle\langle\boldsymbol{n},\boldsymbol{m}|\,\pi^\dagger \tag{41}$$

$$= \sum_M q_M\left|C\left(\frac{n}{2},\frac{n}{2};\frac{m}{2},M-\frac{n}{2};J,M\right)\right|^2\left[\mathcal{N}^2\sum_{\pi,\pi'}\eta_J(\pi^\dagger\pi')\pi'\,|\boldsymbol{n},\boldsymbol{m}\rangle\langle\boldsymbol{n},\boldsymbol{m}|\,\pi^\dagger\right]. \tag{42}$$

Crucially, the state in brackets is independent of M and must therefore be identical to the state we named $|\psi(\boldsymbol{n},\boldsymbol{m},J)\rangle$ in Supplementary Eq. (24). Hence the remaining analysis runs exactly as in the orthogonal spin case, apart from the replacement of p_J by $\sum_M q_M\left|C\left(\frac{n}{2},\frac{n}{2};\frac{m}{2},M-\frac{n}{2};J,M\right)\right|^2$. Thus, all that changes is the probability distribution over J, and this only depends on the probability over M, determined ultimately by the angle θ.

SUPPLEMENTARY NOTE 4 – DIMENSION COUNTING

From [6, Chapter 7], we have (labelling by λ instead of J)

$$\dim\mathcal{H}_x^\lambda = \frac{\prod_{1\le i<j\le d}(\tilde{\lambda}_i-\tilde{\lambda}_j)}{\prod_{m=1}^{d-1} m!}, \tag{43}$$

$$\tilde{\lambda} := \lambda + (d-1,d-2,\ldots,0). \tag{44}$$

First take the bosonic case. Since the Young diagram for the SU(2) spin representation has no more than two rows, the same λ labelling the spatial part has no more than two *non-zero* rows. Hence we have $\tilde{\lambda} = \left(\frac{N}{2}+J+d-1,\ \frac{N}{2}-J+d-2,\ d-3,\ d-4,\ldots 0\right)$. Calculating the product in the numerator of Supplementary Eq. (43) is aided by the table below, which lists the values of $\tilde{\lambda}_i-\tilde{\lambda}_j$, where i labels the row and $j>i$ labels the column:

	2	3	4	5	…	$d-1$	d
1	$2J+1$	$\frac{N}{2}+J+2$	$\frac{N}{2}+J+3$	…	…	…	$\frac{N}{2}+J+d-1$
2		$\frac{N}{2}-J+1$	$\frac{N}{2}-J+2$	…	…	…	$\frac{N}{2}-J+d-2$
3			1	2	…	…	$d-3$
4				1	…	…	$d-4$
⋮							⋮
$d-2$						1	2
$d-1$							1

(45)

The product of the terms in the first row is

$$(2J+1)\frac{(\frac{N}{2}+J+d-1)!}{(\frac{N}{2}+J+1)!}, \tag{46}$$

the second row gives

$$\frac{(\frac{N}{2}-J+d-2)!}{(\frac{N}{2}-J)!}, \tag{47}$$

and the remaining rows give

$$\prod_{m=1}^{d-3} m!. \tag{48}$$

Putting these into Supplementary Eq. (43) results in the expression for $d^B_{N,J}$ in (13)[main text].

For fermions, we instead use the transpose of the Young diagram, with

$$\lambda^T = (\underbrace{2,\ldots,2}_{\frac{N}{2}-J},\underbrace{1,\ldots,1}_{2J}). \tag{49}$$

An important restriction on λ^T is that the number of rows can never be greater than the dimension, so $\frac{N}{2}+J \leq d$. We find

$$\tilde{\lambda^T} = (\underbrace{d+1, d, d-1, \ldots, d-\frac{N}{2}+J+2}_{\frac{N}{2}-J}, \underbrace{d-\frac{N}{2}+J, d-\frac{N}{2}+J-1, \ldots, d-\frac{N}{2}-J+1}_{2J}, \underbrace{d-\frac{N}{2}-J-1, \ldots, 0}_{d-\frac{N}{2}-J}). \tag{50}$$

As before, the differences $\tilde{\lambda^T}_i - \tilde{\lambda^T}_j$ can be arranged as follows:

	2	3	...	$\frac{N}{2}-J$	$\frac{N}{2}-J+1$	$\frac{N}{2}-J+2$	...	$\frac{N}{2}+J$	$\frac{N}{2}+J+1$	$\frac{N}{2}+J+2$	...	$d-1$	d
1	1	2	...	$\frac{N}{2}-J-1$	$\frac{N}{2}-J+1$	$\frac{N}{2}-J+2$	...	$\frac{N}{2}+J$	$\frac{N}{2}+J+2$	$\frac{N}{2}+J+3$	...	d	$d+1$
2		1	...	$\frac{N}{2}-J-2$	$\frac{N}{2}-J$	$\frac{N}{2}-J+1$	...	$\frac{N}{2}+J-1$	$\frac{N}{2}+J+1$	$\frac{N}{2}+J+2$	...	$d-1$	d
$\vdots$				$\vdots$	$\vdots$	$\vdots$		$\vdots$	$\vdots$	$\vdots$		$\vdots$	$\vdots$
$\frac{N}{2}-J-1$				1	3	4	...	$2J+2$	$2J+4$	$2J+5$	...	$d-(\frac{N}{2}-J)+2$	$d-(\frac{N}{2}-J)+3$
$\frac{N}{2}-J$					2	3	...	$2J+1$	$2J+3$	$2J+4$	...	$d-(\frac{N}{2}-J)+1$	$d-(\frac{N}{2}-J)+2$
$\frac{N}{2}-J+1$						1	...	$2J-1$	$2J+1$	$2J+2$	...	$d-(\frac{N}{2}-J)-1$	$d-(\frac{N}{2}-J)$
$\vdots$								$\vdots$	$\vdots$	$\vdots$		$\vdots$	$\vdots$
$\frac{N}{2}+J-1$								1	3	4	...	$d-(\frac{N}{2}+J)+1$	$d-(\frac{N}{2}+J)+2$
$\frac{N}{2}+J$									2	3	...	$d-(\frac{N}{2}+J)$	$d-(\frac{N}{2}+J)+1$
$\frac{N}{2}+J+1$										1	...	$d-(\frac{N}{2}+J)-2$	$d-(\frac{N}{2}+J)-1$
$\vdots$												$\vdots$	$\vdots$
$d-2$												1	2
$d-1$													1

(51)

Here, the bold lines indicate the division into the three main index groups. We want to calculate the product of all rows in the table. The bottom group of rows gives

$$\prod_{m=1}^{d-(N/2+J)-1} m!. \tag{52}$$

The next group up, being careful to discount the terms lost due to the jump at column $j = N/2+J$, gives

$$\prod_{m=d-(N/2+J)+1}^{d-(N/2-J)} \frac{m!}{m-(d-(N/2+J))} \tag{53}$$

Finally, the top group of rows, noting the additional jump at $j = N/2-J+1$, gives

$$\prod_{m=d-(N/2-J)+2}^{d+1} \frac{m!}{[m-(d-(N/2+J))][m-(d-(N/2-J)+1)]}. \tag{54}$$

Inserting into Supplementary Eq. (43), we need to divide the product of the above three terms by $\prod_{m=1}^{d-1} m!$. This factor cancels all the factorials present in the above three expressions, with the exception of the top two rows, and contributes two factorials occurring at $m = d(N/2+J)$, $d-(N/2-J)+1$. Therefore we have

$$d^F_{N,J} = \prod_{r=d-N/2-J+1}^{d-N/2+J} \frac{1}{r-d+N/2+J} \tag{55}$$

$$\cdot \prod_{m=d-N/2+J+2}^{d+1} \frac{1}{(m-d+N/2+J)(m-d+N/2-J-1)} \cdot \frac{d!(d+1)!}{(d-N/2+J+1)!(d-N/2-J)!} \tag{56}$$

$$= \frac{1}{(2J)!} \cdot \frac{(2J+1)!}{(N/2+J+1)!(N/2-J)!} \cdot \frac{d!(d+1)!}{(d-N/2+J+1)!(d-N/2-J)!} \tag{57}$$

$$= \frac{(2J+1)d!(d+1)!}{(N/2+J+1)!(N/2-J)!(d-N/2+J+1)!(d-N/2-J)!}. \tag{58}$$

SUPPLEMENTARY NOTE 5 – LOW DENSITY LIMIT

Bosons

Here we prove equation (23)[main text] for bosons. For simplicity, we take $n = m$. The result rests on the observation that, for sufficiently large d, the ratio $d_J^B/p_J \approx \binom{n+d-1}{n}^2$. We have

$$\frac{d_J^B/p_J}{\binom{n+d-1}{n}^2} = \frac{(d-1)!(d+n+J-1)!(d+n-J-2)!}{(d-2)!(d+n-1)!^2} \tag{59}$$

$$= (d-1)\frac{\prod_{k=0}^{J-1}(d+n+k)}{\prod_{k=0}^{J}(d+n-J-1+k)} \tag{60}$$

$$= \left(1-\frac{1}{d}\right)\prod_{k=0}^{J-1}(1+[n+k]/d)\prod_{k=0}^{J}(1+[n-J-1+k]/d)^{-1} \tag{61}$$

Letting $x_k = [n+k]/d$, we have

$$\prod_{k=0}^{J-1}(1+[n+k]/d) = \sum_{k=0}^{J-1} x_k + \sum_{0=k<l}^{J-1} x_k x_l + O(\epsilon^3) \tag{62}$$

$$= \sum_{k=0}^{J-1} x_k + \frac{1}{2}\left[\left(\sum_{k=0}^{J-1} x_k\right)^2 - \sum_{k=0}^{J-1} x_k^2\right] + O(\epsilon^3) \tag{63}$$

$$=: B_1 + B_2 + O(\epsilon^3), \tag{64}$$

where the first and second order terms are evaluated to be

$$B_1 = \frac{J(2n+J-1)}{2d}, \tag{65}$$

$$B_2 = \frac{J(J-1)(J[12n-7]+12n[n-1]+3J^2+2)}{24d^2}, \tag{66}$$

and $\epsilon = n^2/d$. Similarly, letting $y_k = [n-J-1+k]/d$,

$$\prod_{k=0}^{J}(1+[n-J-1+k]/d) = \sum_{k=0}^{J} y_k + \frac{1}{2}\left[\left(\sum_{k=0}^{J} y_k^2\right)^2 - \sum_{k=0}^{J} y_k^2\right] + O(\epsilon^3) \tag{67}$$

$$=: C_1 + C_2 + O(\epsilon^3), \tag{68}$$

with

$$C_1 = \frac{(J+1)(2n-J-2)}{2d}, \tag{69}$$

$$C_2 = \frac{J(J+1)(12n^2-12n[J+2]+3J^2+11J+10)}{24d^2}. \tag{70}$$

We then have

$$\frac{d_J^B/p_J}{\binom{n+d-1}{n}^2} = \left(1-\frac{1}{d}\right)(1+B_1+B_2)(1+C_1+C_2)^{-1} + O(\epsilon^3) \tag{71}$$

$$= 1 + R_1 + R_2 + O(\epsilon^3), \tag{72}$$

$$R_1 = B_1 - C_1 - \frac{1}{d} \tag{73}$$

$$= \frac{J(J+1)-n}{d}, \tag{74}$$

$$R_2 = B_2 - C_2 + C_1^2 - \frac{B_1}{d} + \frac{C_1}{d} - B_1 C_1 \tag{75}$$

$$= \frac{2n^2 - 2n(2J[J+1]+1) + J(J+1)(J^2+J+2)}{2d^2}. \tag{76}$$

We now use this to compute the deficit in the change of entropy, as compared with the entropy for the informed observer:

$$\Delta S_{\text{igno}} - \Delta S_{\text{info}} = \sum_J p_J \ln\left(\frac{d_x^J/p_J}{\binom{n+d-1}{n}^2}\right) + p_J \ln p_J \tag{77}$$

$$= \sum_J p_J \ln\big(1 + R_1 + R_2 + O[\epsilon^3]\big) - H(\boldsymbol{p}) \tag{78}$$

$$= \sum_J p_J \left(R_1 + R_2 - \frac{R_1^2}{2}\right) + O(\epsilon^3) - H(\boldsymbol{p}), \tag{79}$$

having used the expansion $\ln(1+x) = x - x^2/2 + \dots$ for small x.

In order to compute the first and second order terms in Supplementary Eq. (79) exactly, we need the following sums involving binomial coefficients:

$$\sum_{J=0}^{n} \binom{2n+1}{n+J+1}(2J+1) = (2n+1)\binom{2n}{n}, \tag{80}$$

$$\sum_{J=0}^{n} \binom{2n+1}{n+J+1}(2J+1)J(J+1) = (2n)(2n+1)\binom{2n-1}{n-1}. \tag{81}$$

These are both proved using the easily checked identity

$$\frac{N-2k}{N}\binom{N}{k} = \binom{N-1}{k} - \binom{N-1}{k-1}. \tag{82}$$

For Supplementary Eq. (80), we have (setting $k = n - J$, $N = 2n+1$)

$$\sum_{J=0}^{n} \binom{2n+1}{n+J+1}(2J+1) = \sum_{J=0}^{n} \binom{2n+1}{n-J}(2J+1) \tag{83}$$

$$= \sum_{k=0}^{n} \binom{2n+1}{k}(2n+1-2k) \tag{84}$$

$$= \sum_{k=0}^{n} (2n+1)\left[\binom{2n}{k} - \binom{2n}{k-1}\right] \tag{85}$$

$$= (2n+1)\binom{2n}{n}. \tag{86}$$

Similarly, for Supplementary Eq. (81),

$$\sum_{J=0}^{n} \binom{2n+1}{n+J+1}(2J+1)J(J+1) = \sum_{k=0}^{n} \binom{2n+1}{k}(2n+1-2k)(n-k)(n-k+1) \tag{87}$$

$$= \sum_{k=0}^{n} (2n+1)\left[\binom{2n}{k} - \binom{2n}{k-1}\right](n-k)(n-k+1) \tag{88}$$

$$= (2n+1)\sum_{k=0}^{n} \binom{2n}{k}(n-k)(n-k+1) - (2n+1)\sum_{k=0}^{n-1} \binom{2n}{k}(n-k-1)(n-k) \tag{89}$$

$$= (2n+1)\sum_{k=0}^{n-1} \binom{2n}{k}(n-k)\left[(n-k+1) - (n-k-1)\right] \tag{90}$$

$$= (2n+1)\sum_{k=0}^{n-1} \binom{2n}{k}(2n-2k), \tag{91}$$

and by using Supplementary Eq. (82) with $N = 2n$,

$$\sum_{J=0}^{n} \binom{2n+1}{n+J+1}(2J+1)J(J+1) = (2n+1)(2n)\sum_{k=0}^{n-1} \binom{2n-1}{k} - \binom{2n-1}{k-1} \tag{92}$$

$$= (2n+1)(2n)\binom{2n-1}{n-1}. \tag{93}$$

Recall that

$$p_J = \frac{(n!)^2}{(2n+1)!}\binom{2n+1}{n+J+1}(2J+1), \tag{94}$$

so the first order contribution is

$$\sum_{J=0}^{n} p_J R_1(J) = \sum_{J=0}^{n} p_J \frac{J(J+1)-n}{d} \tag{95}$$

$$= -\frac{n}{d} + \frac{(n!)^2}{d(2n+1)!}\binom{2n+1}{n+J+1}(2J+1)J(J+1) \tag{96}$$

$$= -\frac{n}{d} + \frac{(n!)^2}{d(2n+1)!}(2n+1)(2n)\binom{2n-1}{n-1} \tag{97}$$

$$= -\frac{n}{d} + \frac{(n!)^2(2n+1)(2n)(2n-1)!}{d(2n+1)!(n-1)!(n!)} \tag{98}$$

$$= -\frac{n}{d} + \frac{n}{d} = 0. \tag{99}$$

The second order is

$$\sum_J p_J \left[R_2(J) - \frac{R_1(J)^2}{2}\right] = \sum_{J=0}^{n} p_J \frac{n(n-2) - 2(n-1)J(J+1)}{2d^2} \tag{100}$$

$$= \frac{n(n-2)}{2d^2} - \frac{2(n-1)}{2d^2}\sum_{J=0}^{n} p_J J(J+1) \tag{101}$$

$$= \frac{n(n-2)}{2d^2} - \frac{2(n-1)}{2d^2} n \tag{102}$$

$$= -\frac{n^2}{2d^2}. \tag{103}$$

Therefore, substituting the above into Supplementary Eq. (79), we have

$$\Delta S_{\text{igno}} - \Delta S_{\text{info}} = -H(\boldsymbol{p}) - \frac{n^2}{2d^2} + O\left(\frac{n^3}{d^3}\right). \tag{104}$$

Fermions

The method is the same as for bosons. We expand $\frac{d_J^F/p_J}{\binom{d}{n}^2}$ to second order. Letting $z_k = [k-n-J]/d$, we have

$$\prod_{k=1}^{J}(1 + [k-n-J]/d) = F_1 + F_2 + O(\epsilon^3), \tag{105}$$

where

$$F_1 = \sum_{k=1}^{J} z_k = \frac{-J(2n+J-1)}{2d}, \tag{106}$$

$$F_2 = \frac{1}{2}\left[F_1^2 - \sum_{k=1}^{J} z_k^2\right] = \frac{J(J-1)(2+3J^2+12n[n-1]+J[12n-7])}{24d^2}. \tag{107}$$

Similarly, letting $w_k = [k-n+1]/d$,

$$\prod_{k=0}^{J}(1+[k-n+1]/d) = G_1 + G_2 + O(\epsilon^3), \tag{108}$$

where

$$G_1 = \sum_{k=0}^{J} w_k + \frac{(J+1)(J-2n+2)}{2d}, \tag{109}$$

$$G_2 = \frac{1}{2}\left[G_1^2 - \sum_{k=0}^{J} w_k^2\right] = \frac{J(J+1)(10+11J+3J^2-12n[J+2]+12n^2)}{24d^2}. \tag{110}$$

We then have

$$\frac{d_J^F/p_J}{\binom{d}{n}^2} = \left(1+\frac{1}{d}\right)(1+F_1+F_2)(1+G_1+G_2)^{-1} + O(\epsilon^3) \tag{111}$$

$$= 1 + T_1 + T_2 + O(\epsilon^3), \tag{112}$$

$$T_1 = F_1 - G_1 + \frac{1}{d} \tag{113}$$

$$= \frac{-J(J+1)+n}{d}, \tag{114}$$

$$T_2 = F_2 - G_2 + G_1^2 + \frac{F_1}{d} - \frac{G_1}{d} - F_1 G_1 \tag{115}$$

$$= \frac{2n^2 - 2n(2J[J+1]+1) + J(J+1)(J^2+J+2)}{2d^2}. \tag{116}$$

Note that compared with the boson case, $T_1 = -R_1$, $T_2 = R_2$, thus the first order vanishes and we again have

$$\Delta S_{\text{igno}} - \Delta S_{\text{info}} = -H(\boldsymbol{p}) - \frac{n^2}{2d^2} + O\left(\frac{n^3}{d^3}\right). \tag{117}$$

SUPPLEMENTARY NOTE 6 – ENTROPY $H(\mathbf{p})$ FOR LARGE PARTICLE NUMBER

Here, we evaluate the entropy $H(\boldsymbol{p})$ for large particle number. We take $n = m \gg 1$. Starting from Supplementary Eq. (23), we can rewrite

$$p_J = (2J+1)\frac{(n!)^2}{(2n+1)!}\binom{2n+1}{n+J+1} \tag{118}$$

$$= (2J+1)\frac{(n!)^2 2^{2n+1}}{(2n+1)!} b(n+J+1), \tag{119}$$

where $b(n+J+1) = 2^{-(2n+1)}\binom{2n+1}{n+J+1}$ follows a binomial distribution with $N+1$ trials and a success probability of 1/2.

Using Stirling's approximation in the form $n! = \sqrt{2\pi} n^{n+1/2} e^{-n+O(1/n)}$ [7], we have

$$\frac{(n!)^2}{(2n+1)!} = \frac{n^{2n+1} e^{-2n+O(1/n)}}{\sqrt{2\pi}(2n+1)^{2n+3/2} e^{-2n-1+O(1/n)}} \tag{120}$$

$$= (\sqrt{2\pi}e)\left(\frac{n}{2n+1}\right)^{2n+1} \frac{1}{(2n+1)^{1/2}}[1+O(1/n)] \tag{121}$$

$$= \frac{\sqrt{2\pi}e}{2^{2n+1}\left(1+\frac{1}{2n}\right)^{2n+1}(2n+1)^{1/2}}[1+O(1/n)] \tag{122}$$

$$= \frac{\sqrt{2\pi}e}{2^{2n+1}[e+O(1/n)](2n+1)^{1/2}}[1+O(1/n)] \tag{123}$$

$$= \frac{1}{2^{2n+1}}\sqrt{\frac{2\pi}{2n+1}}[1+O(1/n)]. \tag{124}$$

Using a local version of the central limit theorem [8, Chapter VII, Theorem 1], we can approximate $b(n+J+1)$ by a normal distribution with mean $(2n+1)/2$ and variance $(2n+1)/4$, obtaining

$$p_J = (2J+1)\sqrt{\frac{2\pi}{2n+1}}[1+O(1/n)]\left[\frac{e^{-\frac{(J+1/2)^2}{n+1/2}}}{\sqrt{2\pi(2n+1)/4}} + o(n^{-1/2})\right] \tag{125}$$

$$= (2J+1)\left[\frac{e^{-\frac{(J+1/2)^2}{n+1/2}}}{n+1/2} + o(1/n)\right][1+O(1/n)] \tag{126}$$

$$= (2J+1)\frac{e^{-\frac{(J+1/2)^2}{n+1/2}}}{n+1/2}[1+o(1)][1+O(1/n)] \tag{127}$$

$$= (2J+1)\frac{e^{-\frac{(J+1/2)^2}{n+1/2}}}{n+1/2}[1+o(1)], \tag{128}$$

where $o(f)$ denotes an error term going to zero strictly faster than f. Then

$$\ln p_J = \ln(2J+1) - \ln(n+1/2) - \frac{(J+1/2)^2}{n+1/2} + o(1), \tag{129}$$

so the entropy is approximated by

$$H(\boldsymbol{p}) = -\sum_{J=0}^{n} p_J\left[\ln(2J+1) - \ln(n+1/2) - \frac{(J+1/2)^2}{n+1/2} + o(1)\right] \tag{130}$$

$$= \ln(n+1/2) + o(1) + [1+o(1)]\sum_{J=0}^{n}(2J+1)\frac{e^{-\frac{(J+1/2)^2}{n+1/2}}}{n+1/2}\left[-\ln(2J+1) + \frac{(J+1/2)^2}{n+1/2}\right]. \tag{131}$$

For large n, we expect that the sum can be approximated by an integral. To show this, we can use the simplest version of the Euler-Maclaurin formula:

$$\sum_{J=0}^{n} f(J) = \int_0^n f(x)\,\mathrm{d}x + \int_0^n \left(x - \lfloor x\rfloor - \frac{1}{2}\right) f'(x)\,\mathrm{d}x + \frac{f(0)+f(n)}{2}, \tag{132}$$

$$f(x) := (2x+1)\frac{e^{-\frac{(x+1/2)^2}{n+1/2}}}{n+1/2}\left[-\ln(2x+1) + \frac{(x+1/2)^2}{n+1/2}\right]. \tag{133}$$

Firstly, we have

$$f(0) = \frac{e^{-\frac{1}{4(n+1/2)}}}{n+1/2}\cdot\frac{1}{4(n+1/2)} = O(n^{-2}), \tag{134}$$

$$f(n) = 2e^{-(n+1/2)}\left[-\ln(2n+1) + (n+1/2)\right] = O(ne^{-n}). \tag{135}$$

Along these lines, it is not hard to see that shifting the initial point from $x=0$ to $x=1/2$ leads to an $o(1)$ error, so we change variables to $y = x+1/2$ and let $g(y) := f(y-1/2)$. Additionally, the upper limit can be extended to infinity with an error which can be verified to be $O(e^{-n}\mathrm{poly}[n,\ln n])$. For the remainder integral, we let $k = (n+1/2)^{-1}$ and use

$$g(y) = 2ke^{-ky^2}\left[-y\ln(2y) + ky^3\right], \tag{136}$$

$$g'(y) = 2ke^{-ky^2}\left[2ky^2\ln(2y) - 2k^2y^4 - \ln(2y) - 1 + 3ky^2\right]. \tag{137}$$

Together with $|y - \lfloor y\rfloor - 1/2| \le 1/2$, we have

$$\left|\int_0^\infty \left(y - \lfloor y\rfloor - \frac{1}{2}\right) g'(y)\,\mathrm{d}y\right| \le \left|\int_0^\infty 2k^3 y\ln(2y)e^{-ky^2}\,\mathrm{d}y\right| + \left|\int_0^\infty 2k^3y^4e^{-ky^2}\,\mathrm{d}y\right| + \left|\int_0^\infty k\ln(2y)e^{-ky^2}\,\mathrm{d}y\right| \tag{138}$$

$$+ \left|\int_0^\infty ke^{-ky^2}\,\mathrm{d}y\right| + \left|\int_0^\infty 3k^2y^2e^{-ky^2}\,\mathrm{d}y\right| \tag{139}$$

in which the individual integrals can be evaluated with the highest order being $O\left(\frac{\ln n}{n}\right) = o(1)$.

Overall, therefore,

$$\sum_{J=0}^{n} f(J) = \int_0^\infty g(y)\,\mathrm{d}y + o(1) \tag{140}$$

$$= \frac{1}{2}(\ln k + \gamma) - \ln 2 + 1 + o(1) \tag{141}$$

$$= -\frac{1}{2}\ln n + \frac{\gamma}{2} - \ln 2 + 1 + o(1), \tag{142}$$

where $\gamma = 0.557\ldots$ is the Euler-Mascheroni constant. Putting this into Supplementary Eq. (131),

$$H(\boldsymbol{p}) = \frac{1}{2}\ln n + \frac{\gamma}{2} - \ln 2 + 1 + o(1) \tag{143}$$

$$= \frac{1}{2}\ln n + 0.595... + o(1). \tag{144}$$

[1] Harrow, A. W. Applications of coherent classical communication and the Schur transform to quantum information theory. *arXiv preprint quant-ph/0512255* (2005).

[2] Hamermesh, M. *Group Theory and Its Application to Physical Problems*. Addison Wesley Series in Physics (Dover Publications, 1989).

[3] Biedenharn, L. C. & Louck, J. D. *Angular Momentum in Quantum Physics* (Addison-Wesley, Reading, MA, 1981).

[4] Horodecki, M. & Oppenheim, J. Fundamental limitations for quantum and nanoscale thermodynamics. *Nature communications* **4**, 2059 (2013).

[5] Lostaglio, M., Jennings, D. & Rudolph, T. Description of quantum coherence in thermodynamic processes requires constraints beyond free energy. *Nature Communications* **6**, 6383 (2015).

[6] Goodman, R. & Wallach, N. R. *Symmetry, Representations, and Invariants*, vol. 255 of *Graduate Texts in Mathematics* (Springer New York, New York, NY, 2009).

[7] Flajolet, P. & Sedgewick, R. *Analytic Combinatorics* (Cambridge University Press, Cambridge, 2009).

[8] Petrov, V. V. *Sums of Independent Random Variables* (Springer Berlin Heidelberg, Berlin, Heidelberg, 1975).
